# USE OF DIGITAL TECHNOLOGY SOLUTIONS TO SUPPORT CAREGIVING BY FAMILY CAREGIVERS OF OLDER ADULTS

**DOI:** 10.1093/geroni/igac059.349

**Published:** 2022-12-20

**Authors:** Rahul Malhotra, Nur Diyana Azman, Veronica Shimin Goh, Abhijit Visaria

**Affiliations:** Duke-NUS Medical School, Singapore, Singapore; Duke-NUS Medical School, Singapore, Singapore; Duke-NUS Medical School, Singapore, Singapore; Duke-NUS Medical School, Singapore, Singapore

## Abstract

Caregiving-relevant information and services are increasingly available online. Greater understanding of the extent and purpose of their use by family caregivers of older adults and specific caregiver sub-groups that are more or less likely to use them can inform both policies related to and the content of such solutions. We investigated the extent to which family caregivers of older persons in Singapore use digital technology solutions such as the internet and apps, and the purposes for which they use them. Information on digital device use was collected from 278 caregivers. Of them, 139 caregivers gave detailed information on how they had used the internet or apps to support caregiving in the last six months. Most (89%) caregivers used digital devices regularly (mostly smartphones (87%)). Digital device use was associated with caregiver age, ethnicity and education. Common generic online activities included sending instant messages (77%) and surfing websites (64%). While 54% had used the internet to support caregiving (most common purpose: search for information on care-recipient’s health conditions), 43% had used apps to do so (most common purpose: coordinate care with family members or other caregivers). Such use was associated with caregiver age, education and care-recipient health. While use of digital devices and generic online activities are common among caregivers, their use of the internet or apps to support caregiving is less common. Information-seeking and coordination are indicative of avenues in which digital technology solutions can complement ‘physical’ channels of communication and support with and for caregivers, and be further expanded.

